# Drugs in the environment, viruses in the balance: a review of the untold nexus of pharmaceuticals and virology

**DOI:** 10.3389/fpubh.2026.1926350

**Published:** 2026-09-03

**Authors:** Ahmed Abd Zaid, Allal Ouhtit

**Affiliations:** 1 College of Arts and Sciences, Department of Applied & Natural Sciences, American University of Iraq-Baghdad, Baghdad, Iraq; 2 College of Health Sciences, American University of Iraq-Baghdad, Baghdad, Iraq

**Keywords:** bacteriophages, ecotoxicology, environmental risk assessment, environmental virology, pharmaceutical pollution, viral ecology

## Abstract

**Objectives:**

The pervasive contamination of aquatic and terrestrial ecosystems by active pharmaceutical ingredients (APIs) is a well-documented environmental challenge. However, the impact of this pollution on the most abundant biological entities on Earth viruses has remained a critical blind spot. This review provides the first integrated synthesis of the bidirectional nexus between pharmaceutical pollution and environmental virology.

**Methods:**

A comprehensive literature search was conducted in PubMed, Web of Science, and Scopus using search terms related to pharmaceuticals, viruses, and the environment. Additional targeted searches were performed for specific drug classes and viral systems. Articles were selected based on their relevance to pharmaceutical-viral ecology interactions.

**Results:**

We introduce a novel dual-pathway framework positing that APIs influence viral ecology through: (i) direct “drug-to-virus” interactions, where non-antibiotic compounds can physiochemically enhance viral stability and infectivity, and (ii) indirect “drug-to-host-to-virus” pathways, where antibiotics stress bacterial hosts, triggering prophage induction and fundamentally altering viral community dynamics. These mechanisms have profound implications, including the phage-mediated dissemination of antibiotic resistance genes, the selection for antiviral resistance in environmental reservoirs, and the potential for immunosuppression in aquatic fauna. Extending this framework to emerging biopharmaceuticals, we examine the environmental fate of vaccine nanoparticles and the ecological risks posed by gene therapy viral vectors.

**Conclusion:**

We propose a next-generation environmental risk assessment paradigm that integrates viral ecological outcomes, moving beyond traditional chemical-focused models. This review advances the field from fragmented evidence to a unified framework for environmental public health policy and practice.

## 1 Introduction

Ecotoxicological risk assessment of pharmaceutical pollution has traditionally focused on bacteria, algae, and higher organisms, leaving the most abundant biological entities on Earth viruses as a critical blind spot (1–5). The pervasive presence of active pharmaceutical ingredients (APIs) in global aquatic and terrestrial ecosystems is a defining challenge of the Anthropocene (1, 2). Originating from patient excretion, improper disposal of unused medications, and effluence from manufacturing facilities, these persistent pollutants often bypass conventional wastewater treatment processes, leading to their continual introduction into the environment (3, 4). Consequently, APIs are now routinely detected in surface waters, groundwater, and even drinking water, creating complex mixtures of biologically active compounds at low but constant concentrations (5). The ecotoxicological consequences are increasingly documented, with various drug classes linked to adverse effects such as altered behavior, impaired reproduction, and increased mortality in non-target aquatic fauna (6). A primary concern has been the role of antibiotics in driving the selection for antimicrobial resistance (AMR) in bacterial populations, a critical global health threat (7, 8).

However, the narrative of pharmaceutical impact remains incomplete without considering viruses. Bacteriophages (phages), which specifically infect bacteria, are ubiquitous regulators of microbial ecosystems, influencing bacterial population dynamics, global biogeochemical cycles, and evolutionary processes (9, 10). Their role as key vectors for horizontal gene transfer (HGT) is particularly salient in the context of AMR. Through transduction, phages can package and transfer antibiotic resistance genes (ARGs) between bacterial hosts, serving as a major reservoir and dissemination vehicle for genetic resistance (11, 12). Critically, anthropogenic activities, including the widespread release of antibiotics, are strongly correlated with the enrichment of ARGs within viral populations in the environment, suggesting that pharmaceutical pollution actively shapes this viral-mediated resistance pool (13, 14).

While previous reviews have separately addressed pharmaceutical ecotoxicity (1, 5, 7) and environmental virology (9, 10, 12), the bidirectional interactions between these fields remain largely unexplored and synthesized. From an ecotoxicological perspective, this gap is particularly consequential because viruses despite their abundance and ecological significance are systematically excluded from current environmental risk assessment frameworks. Beyond selecting resistant bacteria, antibiotics and other APIs can directly interfere with the delicate virus-host balance. For instance, sub-lethal antibiotic stress can trigger the induction of prophages dormant viral DNA integrated into bacterial chromosomes leading to widespread bacterial lysis and the release of new virions, a process that can simultaneously alter microbial community structure and amplify the transduction of genetic material (15, 16).

This review addresses this critical gap in ecotoxicology by providing the first integrated synthesis of the pharmaceutical-pollution-viral-ecology nexus. We introduce a novel conceptual framework positing that APIs influence viral ecology through two primary, interconnected pathways: (i) the direct “drug-to-virus” pathway, where non-antibiotic APIs can physiochemically interact with viral particles to enhance their stability or infectivity, and (ii) the indirect “drug-to-host-to-virus” pathway, where pharmaceuticals, particularly antibiotics, stress bacterial hosts and disrupt lysogeny, triggering prophage induction and fundamentally altering viral community dynamics. These interactions have the potential to destabilize foundational microbial ecosystems, accelerate the horizontal transfer of genetic resistance, and pose unforeseen risks to public health yet they are systematically excluded from current environmental risk assessment (ERA) frameworks.

This review advances the field of ecotoxicology by:

1. Synthesizing disparate evidence from clinical pharmacology, environmental microbiology, and ecotoxicology into a unified framework.
2. Providing the first consolidated data tables linking specific APIs to documented viral ecological effects and environmental concentrations.
3. Extending the analysis to emerging biopharmaceuticals (vaccine nanoparticles, gene therapy viral vectors) and their potential environmental implications.
4. Proposing a next-generation ecotoxicological risk assessment paradigm that explicitly incorporates viral ecological outcomes, moving beyond traditional chemical-focused assessment.

Herein, we comprehensively review the literature on this untold nexus. We begin by outlining the sources and pathways of pharmaceutical pollution before delving into the mechanisms of direct and indirect viral interactions. We then explore the implications for antiviral drug resistance and the challenges posed by complex environmental mixtures. Finally, we discuss the critical gaps in current ERA frameworks and propose future research directions to integrate viral dynamics into our understanding of pharmaceutical pollution.

## 2 Methods

This review is a narrative review designed to synthesize evidence on the bidirectional nexus between pharmaceutical pollution and environmental virology. A narrative review approach was chosen because the field is interdisciplinary, emerging, and characterized by heterogeneous study designs that do not readily lend themselves to quantitative meta-analysis or systematic review methodologies. The goal is to provide a conceptual synthesis and identify knowledge gaps, rather than to conduct a quantitative summary of effect sizes.

### 2.1 Review questions

The review was guided by the following primary questions:

1. How do active pharmaceutical ingredients (APIs) directly interact with viral particles to alter their stability, persistence, or infectivity?
2. How do APIs indirectly influence viral ecology through effects on bacterial hosts (e.g., prophage induction) or higher organisms (e.g., immunosuppression)?
3. What are the ecological and public health consequences of pharmaceutical–viral interactions, including the spread of antimicrobial resistance?
4. How do emerging biopharmaceuticals (vaccine nanoparticles, gene therapy vectors) pose novel environmental risks?
5. What modifications to current environmental risk assessment (ERA) frameworks are needed to incorporate viral outcomes?

### 2.2 Search strategy

A systematic literature search was conducted in [PubMed / Web of Science] for articles published up to 2025. The following search string was used (adapted for each database):

*(“pharmaceutical*” OR “active pharmaceutical ingredient*” OR “API*” OR “antibiotic*” OR “antiviral*” OR “antidepressant*” OR “NSAID*”) AND (“virus*” OR “viral” OR “bacteriophage*” OR “phage*” OR “virome” OR “viral ecology”) AND (“environment*” OR “aquatic” OR “wastewater” OR “surface water” OR “sediment” OR “soil”) *.

Additional targeted searches were conducted for specific drug classes (e.g., sertraline, ciprofloxacin, oseltamivir, diclofenac) and specific viral systems (e.g., bacteriophage ΦX174, influenza virus, SARS-CoV-2). Reference lists of key reviews and included articles were hand-searched for additional relevant studies.

### 2.3 Inclusion and exclusion criteria

Articles were included if they:

- Were published in peer-reviewed journals.
- Reported original data or comprehensive reviews on pharmaceutical–virus interactions in environmental or laboratory settings.
- Provided quantitative data on environmental concentrations, viral stability/infectivity, or ecological outcomes.
- Were published in English.

Articles were excluded if they:

- Focused exclusively on clinical or therapeutic applications without any environmental relevance.
- Were conference abstracts, editorials, or non-English publications without English abstracts.
- Were unpublished or preprint manuscripts.

### 2.4 Data extraction and synthesis

From eligible articles, we extracted information on: pharmaceutical identity and concentration, virus/system studied, experimental design, key findings, and environmental relevance. As this is a narrative review, no formal quality assessment or risk-of-bias evaluation was performed. This decision is consistent with the review’s aim of providing a conceptual synthesis and identifying research gaps in an emerging field rather than conducting a quantitative meta-analysis. As this is a narrative review, no formal quality assessment or risk-of-bias evaluation was performed. This decision is consistent with the review’s aim of providing a conceptual synthesis and identifying research gaps in an emerging field, rather than conducting a quantitative meta-analysis. The field is characterized by heterogeneous study designs ranging from controlled laboratory binding assays to environmental monitoring studies that do not readily lend themselves to a single quality assessment tool. However, we applied several informal considerations to ensure the reliability of the synthesized evidence. Studies were only included if they were published in peer-reviewed journals, reported original data or comprehensive reviews, and provided quantitative data on environmental concentrations, viral stability/infectivity, or ecological outcomes. Additionally, we prioritized studies with clear experimental designs, reproducible methodologies, and environmentally relevant conditions where available. Clinical or therapeutic studies were only included when they provided mechanistic insights that could be extrapolated to environmental contexts, and such extrapolations are explicitly identified as such in the manuscript (see Section 2.5). Data were synthesized into a narrative framework structured around direct (“drug-to-virus”) and indirect (“drug-to-host-to-virus”) mechanistic pathways, with supporting evidence summarized in tabular format (Tables 1, 2, see Results sections).

**Table 1:** Documented examples of pharmaceutical-virus interactions.

Pharmaceutical (Class)	Concentration/dose	Exposure duration	Viral system	Endpoint/effect	Type of interaction	Evidence type	Environmental relevance	Reference
Diphenhydramine (Antihistamine)	Not applicable (binding study)	*In vitro* binding assay	Ebola Virus (Filovirus)	Inhibition of viral fusion and entry	Direct: Binds to viral glycoprotein (GP1/GP2 interface)	Mechanistic Laboratory	Demonstrates capacity for direct viral protein interaction; environmental extrapolation requires further study	(29)
Sertraline (SSRI)	K_D = 46.5 nM	In vitro binding assay	SARS-CoV-2	Inhibition of ACE2 receptor interaction	Direct: Binds strongly to Spike protein RBD	Mechanistic Laboratory	Demonstrates capacity for direct capsid interaction; potential for environmental stabilization	(30)
Sertraline (SSRI)	Not specified	In vitro	Enterovirus 71 (EV71)	Disruption of pH-dependent viral uncoating	Indirect: Alkalizes host endo-lysosomes	Mechanistic Laboratory	Host-directed effect; relevance to environmental bacteria requires further study	(31)
Sertraline (SSRI)	Not specified	*In vivo* (mouse model)	Influenza A (H1N1)	Reduced immunopathology, improved survival	Indirect: Modulates host immune response (IFN-α, TNF-α, IL-6, IL-10)	Mechanistic Laboratory	Demonstrates immunomodulatory capacity; environmental relevance to aquatic organisms is speculative	(32)
Sertraline & Diphenhydramine (Mixture)	Environmentally relevant doses (e.g., 4.4 μg/kg in fish feed)	28 days	Crucian Carp (Aquatic host)	Oxidative stress, neurotoxicity, behavioral alterations	Indirect: DPH increases SER accumulation, leading to host stress	Mechanistic Laboratory	Direct environmental relevance: demonstrates host susceptibility modulation in aquatic organisms	(33)
Ciprofloxacin (Antibiotic)	Sub-inhibitory concentrations	Variable	Lysogenic Bacteria	Prophage induction, bacterial lysis, viral release	Indirect: Host-stress mediated induction	Direct Environmental	Classic example of environmentally relevant antibiotic-induced prophage induction	(26)

**Table 2:** Documented environmental concentrations of select pharmaceuticals and associated ecological effects.

Pharmaceutical (Class)	Environmental concentration (Matrix)	Exposure duration	Viral system/host	Endpoint/effect	Relevance to viral ecology	Evidence type	Reference
Oseltamivir Carboxylate (Antiviral)	μg/L in STP effluent and river water (seasonal outbreaks)	Continuous environmental exposure	Influenza viruses in waterfowl	Selection of oseltamivir-resistant strains	Direct Pathway & Resistance Selection: Creates selective pressure for resistant variants in environmental reservoirs	Direct Environmental	(47)
Sertraline (SSRI Antidepressant)	Detected in aquatic environments; tested at 4.4 μg/kg in fish feed	28 days	Rainbow Trout (*Oncorhynchus mykiss*)	Behavioral alterations, immunosuppression, hematological changes	Host Susceptibility: Physiological compromise could increase susceptibility to viral pathogens	Mechanistic Laboratory	(64, 73)
Ciprofloxacin (Fluoroquinolone Antibiotic)	25–64 ng/L (PNEC); frequently exceeded in global rivers	Continuous environmental exposure	Environmental bacteria	Selective pressure for AMR, toxicity to cyanobacteria	Indirect Pathway: Classic driver of prophage induction promoting viral-mediated ARG transfer	Direct Environmental	(64, 65)
NSAIDs (e.g., Ibuprofen, Diclofenac)	ng/L to >10 μg/L in surface waters	Continuous environmental exposure	Aquatic organisms (fish)	Immunosuppression, genotoxicity, lysosomal destabilization	Host Susceptibility & Immunomodulation: Immunosuppressive effects may increase viral susceptibility	Mechanistic Laboratory	(66)

### 2.5 Evidence classification and interpretation

To address the interdisciplinary and emerging nature of this field, we adopted a hierarchical framework for classifying evidence. This framework explicitly distinguishes between:

1. Direct environmental evidence: Findings derived from field studies, environmental monitoring, or laboratory experiments conducted under environmentally relevant conditions (e.g., drug concentrations matching those detected in surface waters). This represents the strongest level of evidence for environmental risk assessment.
2. Mechanistic laboratory evidence: Findings from controlled laboratory studies that demonstrate a plausible mechanism of interaction between a pharmaceutical and a virus or host (e.g., binding affinity studies, cell culture experiments). While these studies provide critical mechanistic insights, they do not necessarily confirm that the same interaction occurs in environmental settings at relevant concentrations.
3. Extrapolation or hypothesis: Reasoned extensions of mechanistic evidence to environmental contexts. These represent hypotheses that require further empirical testing in environmentally relevant systems.

Throughout the manuscript, we explicitly indicate the level of evidence supporting each claim. Where the evidence is mechanistic or speculative, we use language such as “demonstrates a capacity for,” “suggests,” or “raises the possibility that,” distinguishing it from direct environmental evidence. This approach ensures transparency and prevents over-interpretation of laboratory findings as established environmental effects.

## 3 Pharmaceutical pollution and viral ecology

The widespread release of active pharmaceutical ingredients (APIs) into the environment poses a growing threat to ecosystem integrity, with emerging consequences for viral ecology. APIs enter environmental matrices through multiple pathways, including human and animal excretion, improper disposal of unused medications, and effluent discharges from manufacturing facilities (17). Despite the scale of this problem, a major regulatory gap persists. There is no cohesive global framework governing pharmaceutical waste production and disposal. Public mishandling of unused drugs remains particularly problematic; for example, a UNESCO/HELCOM report identifies wastewater as the principal source of pharmaceutical contamination in the Baltic Sea, highlighting the transboundary nature of this challenge (18).

Beyond their role as pathogens, viruses are increasingly recognized as innovative bioindicators for pharmaceutical pollution. Sub-lethal concentrations of pharmaceuticals can alter bacterial host physiology and population density, directly affecting bacteriophage (virus–bacteria) replication and stability. Monitoring phage responses in laboratory assays or wastewater samples offers a sensitive, functional assessment of pharmaceutical bioactivity that complements traditional chemical analyses (19).

### 3.1 Sources and pathways of pharmaceutical pollution

Pharmaceuticals are designed for high biological activity and metabolic stability, meaning that substantial proportions of administered APIs are excreted unchanged or as active metabolites into wastewater (19). Their persistence, coupled with diverse emission routes, creates a complex challenge for environmental management. Contamination arises from both diffuse and point sources:

- Diffuse sources:
- *Human and animal excretion*: APIs eliminated via urine and feces enter municipal wastewater systems, while veterinary drugs in intensive livestock operations accumulate in manure. The application of manure as fertilizer provides a direct pathway for leaching into groundwater or runoff into surface waters (19, 20).
- *Improper disposal*: Discarding unused or expired drugs via toilets or household trash introduces concentrated pharmaceutical residues into sewage systems or landfills, with eventual migration into aquatic environments (19, 21).
- Point sources:
- *Manufacturing effluents: Facilities in major production hubs (*e.g.*, India and China)* have been documented releasing wastewater with extremely high API concentrations, often exceeding the assimilative capacity of receiving environments (21).
- *Healthcare facilities*: Hospitals and clinics act as aggregated sources of API-rich effluent derived from patient excretion and disposal practices. Conventional wastewater treatment plants (WWTPs), not designed to remove these complex compounds, often allow pharmaceuticals to bypass treatment and enter rivers, lakes, and estuaries (19, 22).

The convergence of these pathways ensures a continuous infusion of pharmaceuticals into ecosystems, amplifying risks for viral ecology, and necessitating closer examination of their environmental fate.

### 3.2 Types of pharmaceuticals detected in the environment

Pharmaceutical residues are now widely documented in surface waters, groundwater, and even treated drinking water. Their sustained release and incomplete removal during treatment contribute to a state of *pseudo-persistence*, whereby biologically active compounds remain continuously present in the environment (19).

Detected compounds span diverse therapeutic classes, each with distinct environmental persistence, bioactivity, and ecological risks. Pharmaceuticals detected in aquatic environments span multiple classes, including antibiotics (e.g., sulfamethoxazole, ciprofloxacin), non-steroidal anti-inflammatory drugs (e.g., ibuprofen, diclofenac), endocrine disruptors (e.g., 17α-ethinylestradiol), antiretrovirals (e.g., tenofovir), antineoplastics (e.g., cisplatin), and psychoactive drugs (e.g., carbamazepine, fluoxetine) (18, 20, 22–24). These compounds originate from diverse sources including patient excretion, hospital waste, aquaculture, and improper disposal, and their presence in aquatic ecosystems raises concerns ranging from antimicrobial resistance and toxicity to neurobehavioral effects in non-target organisms (18, 20, 22–24).

Among the most ubiquitous contaminants are non-steroidal anti-inflammatory drugs (NSAIDs) and analgesics, including ibuprofen, diclofenac, naproxen, and acetaminophen. Their widespread over-the-counter use and incomplete metabolism lead to frequent discharge into sewage systems and consistent detection in aquatic environments worldwide (19, 23).

Antibiotics, including sulfonamides, tetracyclines, fluoroquinolones, and nitroimidazoles, represent a class of a particular ecological and public health concern. Their persistence exerts strong selective pressure on microbial communities, driving the enrichment of antimicrobial resistance (AMR) genes (19, 21). Similarly, endocrine disrupting compounds (EDCs), such as natural and synthetic hormones, routinely evade conventional wastewater treatment. Even at trace concentrations, they impair reproduction and disrupt endocrine function in aquatic organisms (19).

The global scale-up of antiretroviral therapy (ART) for HIV has led to rising concentrations of antiretroviral drugs (ARVs) in wastewater, raising questions about their potential role in selecting for resistant viral strains (19). Anti-neoplastic agents, designed for cytotoxicity, are often excreted in active forms and pose additional hazards, including genotoxic, mutagenic, and teratogenic effects in non-target organisms (19, 24). Beta-blockers (e.g., propranolol) and psychoactive drugs (e.g., carbamazepine) are also of concern due to their persistence in wastewater treatment plants (WWTPs). Chronic, low-level exposure in aquatic systems has been linked to sublethal neurotoxic and reproductive effects that destabilize populations (19, 25).

The consistent co-occurrence of these diverse pharmaceutical classes creates a complex mixture of contaminants, whose cumulative ecological impacts remain poorly understood and demand further investigation.

### 3.3 Mechanisms of interaction: direct and indirect pathways

The environmental presence of Active Pharmaceutical Ingredients (APIs) can inadvertently influence viral communities through two primary mechanistic pathways: the direct “drug-to-virus” pathway, where the API interacts with the viral particle itself, and the indirect “drug-to-virus” pathway, where the API’s effect on a host organism subsequently alters viral dynamics (26). The key mechanistic evidence for these pathways, drawn from both clinical and environmental studies, is summarized in Table 1, which provides specific examples of pharmaceuticals, their viral targets or hosts, and the nature of their interaction. The Direct “Drug-to-Virus” Pathway.

#### 3.3.1 The direct “drug-to-virus” pathway

This pathway involves direct chemical or physical interaction between an API and a viral particle in the environment, potentially altering viral stability, integrity, or infectivity (27). This approach is exemplified by the known mechanism of many antiviral drugs, which target viral components like glycoproteins or enzymes (28). The ecological concern is that these same mechanisms, when triggered unintentionally in the environment, can disrupt viral ecology. (i) *Diphenhydramine and Viral Glycoproteins:* Diphenhydramine (DPH), a common antihistamine, has been identified as a potential anti-filovirus agent through direct interaction with the Ebola virus glycoprotein (EBOV-GP) (29). This demonstrates a proven capacity for DPH to bind directly to viral surface proteins. In an environmental context, this binding potential raises a critical question: could DPH, at sub-therapeutic concentrations in water systems, similarly interact with surface proteins of environmentally relevant viruses, such as bacteriophages, thereby stabilizing them or altering their infectivity? This hypothesis moves the direct “drug-to-virus” pathway from theory to a plausible, mechanism-driven phenomenon. (ii) *Sertraline and Viral Structural Proteins:* Sertraline (SRT), a selective serotonin reuptake inhibitor (SSRI), demonstrates direct antiviral activity against SARS-CoV-2 by targeting its spike (S) protein (30). This interaction, characterized by a strong binding affinity, suggests that SRT has an inherent capacity to interact with and stabilize viral capsid proteins. In aquatic environments, such a stabilizing interaction could directly prolong the environmental persistence and infectious window of a range of viruses, representing a clear “drug-to-virus” effect with significant ecological implications.

#### 3.3.2 The indirect “drug-to-host-to-virus” pathway

The indirect “drug-to-host-to-virus” pathway involves targeting host cellular factors or pathways that the virus relies on (26). In an environmental context, this occurs when an API exerts stress on a host organism, which in turn influences the outcome of viral infection. This strategy can alter the host cell’s susceptibility or provoke a viral response from a dormant state. (i) *Sertraline and Host Compartment Alkalization:* Sertraline exhibits an indirect effect against Enterovirus 71 (EV71) by accumulating in and alkalizing acidic intracellular compartments like endosomes and lysosomes (31). Since the entry of many viruses is a pH-dependent process, this alteration of the host cell environment directly interferes with viral uncoating. In environmental bacteria, a similar disruption of internal pH by accumulating pharmaceuticals could indirectly prevent or alter the infection cycle of bacteriophages that rely on pH-dependent entry mechanisms. (ii) *Antibiotic-Induced Prophage Induction:* A classic example of this pathway is the triggering of the lytic cycle in lysogenic bacteria by antibiotics. When a bacterial host is stressed by an antibiotic, the dormant prophage (viral DNA) can become activated, leading to viral replication, host cell lysis, and the release of new virions. This process, a form of host-directed viral activation, can be triggered by various antibiotic classes and profoundly amplifies viral particles and gene transfer in environmental settings. (iii) *Immunomodulation and Ecotoxicity:* Sertraline demonstrates an indirect immunomodulatory effect in influenza A H1N1 infection by regulating pro-inflammatory cytokines, thereby mitigating severe pathology (32). Furthermore, in an ecological context, the combined exposure of aquatic organisms like crucian carp to SRT and DPH leads to severe biochemical and behavioral alterations (33). A physiologically compromised host, suffering from oxidative stress and neurotoxicity, may exhibit a weakened immune response or altered cell surfaces, potentially increasing its susceptibility to viral pathogens. This illustrates a complex indirect pathway where pharmaceuticals modify the host’s health, which in turn can modulate its virome and susceptibility to viral diseases.

**Figure 1:**
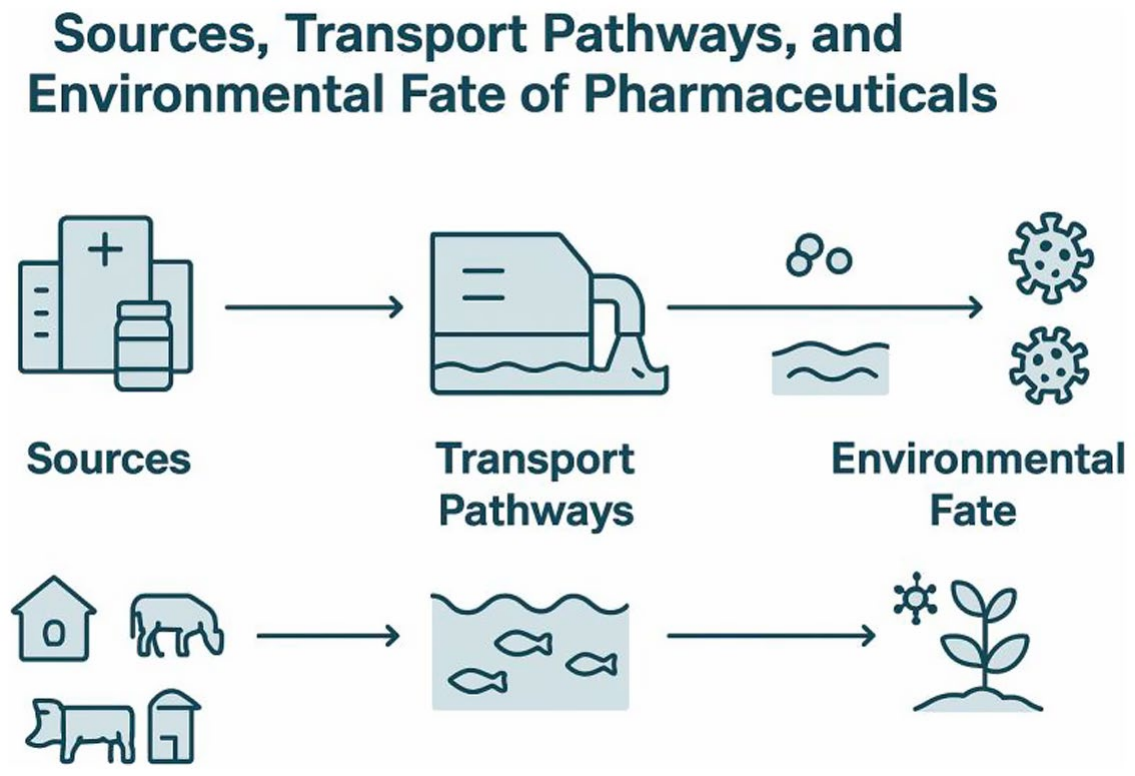
The pathways of pharmaceutical pollution.

The complex interplay between pharmaceutical pollution and viruses, encompassing the direct ‘drug-to-virus’ and indirect ‘drug-to-host-to-virus’ pathways, is summarized in Figure 1. This conceptual model integrates the mechanisms detailed above, illustrating how APIs from various sources can ultimately impact viral stability, infectivity, and evolution, with cascading effects on microbial communities and public health.

In conclusion, the interplay between pharmaceutical pollution and viruses in the environment is governed by these direct and indirect pathways. Evidence from both clinical repurposing studies and environmental ecotoxicology confirms that APIs are not inert in ecosystems but can actively reshape viral ecology, with profound implications for microbial community structure, genetic transfer, and overall ecosystem health.

#### 3.3.3 Evidence for environmental relevance of direct drug-virus interactions

A central question underlying the pharmaceutical-virology nexus is whether APIs are present in the environment at concentrations sufficient to directly alter viral properties. Environmental monitoring data confirm that multiple pharmaceutical classes are routinely detected in surface waters, wastewater, and groundwater at concentrations ranging from ng/L to low μg/L levels (2, 3). Several lines of evidence support the environmental relevance of direct drug-virus interactions.

Oseltamivir carboxylate, the active metabolite of the antiviral prodrug oseltamivir phosphate (Tamiflu), has been detected in sewage treatment plant effluent and river water at μg/L concentrations during seasonal influenza outbreaks (5). Concurrent studies have documented the selection of oseltamivir-resistant influenza viruses in waterfowl exposed to these environmental concentrations, demonstrating that environmentally relevant levels can exert direct selective pressure on viral populations in natural reservoirs (30). This represents the strongest direct evidence for environmental drug-virus interactions.

Sertraline, an SSRI antidepressant, has been detected in aquatic environments at concentrations that, in laboratory studies, have been shown to bind directly to viral capsid proteins and modulate viral entry mechanisms (30, 33). While direct environmental confirmation of this interaction is pending, the convergence of environmental detection data and mechanistic evidence makes this a plausible pathway. The demonstrated immunosuppressive effects of sertraline in aquatic organisms further suggest that environmentally relevant concentrations can compromise host defenses, indirectly influencing viral susceptibility (24, 25).

Diphenhydramine, an antihistamine, has been shown in laboratory studies to bind directly to viral glycoproteins, demonstrating a capacity for direct viral interaction (29). Environmental monitoring has detected diphenhydramine in surface waters, though its direct effects on environmental viral communities remain a hypothesis requiring further testing (33).

Sub-therapeutic concentrations: A critical consideration is that APIs in the environment are typically present at concentrations far below clinical therapeutic levels. However, sub-therapeutic concentrations can still exert biological effects, including selection for resistance and modulation of host-pathogen interactions (26). This is well-established for antibiotics in the context of antimicrobial resistance selection (28) and is increasingly recognized for antivirals in environmental reservoirs (30).

Classification of evidence: It is important to distinguish between the strength of evidence across these examples. The evidence for oseltamivir resistance selection in waterfowl represents direct environmental evidence. The evidence for sertraline and diphenhydramine interactions is mechanistic laboratory evidence with plausible environmental extrapolation. The potential for these interactions to occur at sub-therapeutic concentrations remains an area of active investigation and hypothesis generation.

In summary, while direct evidence for environmental drug-virus interactions remains limited to a few well-documented cases, the combination of environmental detection data, mechanistic laboratory studies, and documented resistance selection in wildlife provides a compelling case for the environmental relevance of this nexus. The evidence is strongest for antiviral resistance selection (oseltamivir) and indirect host-mediated effects (antibiotic-induced prophage induction), while direct drug-virus interactions represent an emerging area requiring further investigation.

### 3.4 Ecological and evolutionary consequences of viral modulation

The direct and indirect interactions between pharmaceuticals and viruses, as detailed in the previous section, have profound and tangible consequences that cascade across multiple levels of biological organization, from genetic exchange to global ecosystem function.

The impacts on Microbial Community Structure and Function, the indirect, host-mediated pathway, particularly prophage induction, act as a powerful force in restructuring microbial communities. The targeted lysis of dominant bacterial strains creates ecological niches, shifting the taxonomic and functional diversity of the microbiome (34). Furthermore, this widespread bacterial mortality through the “viral shunt” diverts significant amounts of carbon and nutrients away from the classic food web into the dissolved organic matter pool, directly influencing aquatic biogeochemical cycles and ecosystem productivity (35).

Acceleration of Horizontal Gene Transfer and Resistance, dissemination of Pharmaceutical Pollution synergistically amplifies the spread of genetic material. Bacteriophages, key vectors for horizontal gene transfer, see their role dramatically enhanced. Antibiotic stress not only enriches antibiotic-resistant bacteria (ARB) but also simultaneously increases the packaging and transmission of antibiotic resistance genes (ARGs) via transduction (34).

This creates a dangerous feedback loop; pharmaceuticals select for resistance and then promote the mechanism that disseminates it. The concurrent release of virulence factors through this pathway further compounds the public health risk.

Alteration of Viral Evolutionary Trajectories, the selective pressures exerted by pharmaceuticals drive rapid co-evolution. As antibiotics enrich for ARB, bacteriophages must evolve novel strategies to infect these newly dominant, resistant hosts, directly shaping viral evolution in real-time (36). Moreover, sub-lethal concentrations of APIs that directly interact with viral particles whether stabilizing capsids or modulating infectivity present a novel and poorly understood evolutionary pressure, potentially selecting for viral variants that can tolerate or exploit these chemical challenges (37).

Implications for Public Health and Water Treatment, the consequences of this nexus pose direct challenges to human health. The potential for APIs to enhance viral environmental persistence or alter infectivity can compromise the efficacy of water treatment processes designed to inactivating pathogens (38).

The enrichment of ARGs and virulence factors in environmental viral reservoirs increases the genetic raw material available for the emergence of future pathogens, representing a significant and long-term One Health risk.

**Figure 2:**
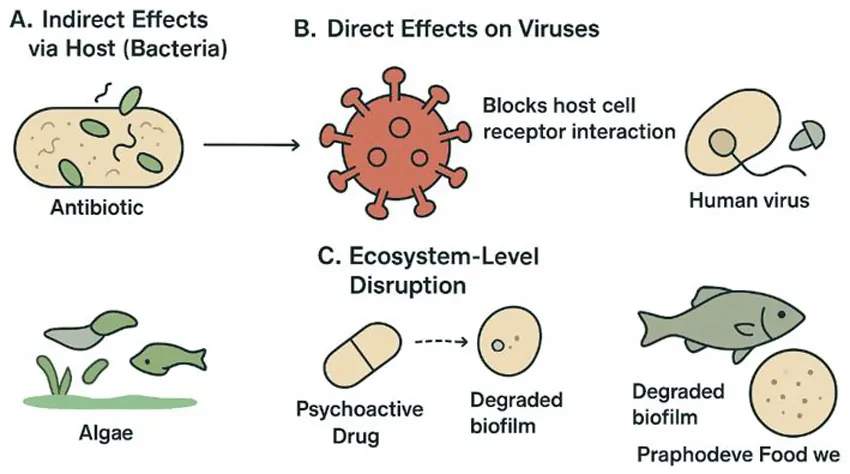
Mechanisms and ecological consequences of pharmaceutical-virus interactions.

In summary, pharmaceutical pollution acts as a disruptive force on the viral engines that drive microbial evolution and ecosystem function. The mechanisms detailed in Section 2.3 lead to consequences that cascade from the molecular level to global public health, as synthesized in Figure 2.

Thus, pharmaceutical pollution presents a multifaceted challenge, influencing viral evolution, host-virus interactions, and ecosystem-scale microbial networks through interconnected direct and indirect pathways.

The mechanistic and ecological relationships discussed in sections 2.3 and 2.4 are underpinned by the confirmed environmental presence of APIs at biologically active concentrations. The widespread detection of these compounds in aquatic systems provides the essential context for assessing the real-world relevance of the pharmaceutical-virology nexus. Table 2 summarizes environmental monitoring data for key pharmaceuticals, linking their measured concentrations to documented ecological effects and their specific implications for viral ecology.

## 4 Environmental virology as a tool in mitigating pharmaceutical pollution

The widespread occurrence of pharmaceuticals in global water bodies is a well-documented environmental challenge (39, 40), driving the need for innovative remediation strategies. In this context, the field of environmental virology offers a visionary, though largely untested, toolkit. Among the most promising conceptual approaches is the targeted use of bacteriophages (phages). However, translating these concepts from the laboratory to real-world application presents profound challenges that must be critically evaluated (41, 42).

### 4.1 Conceptual pathways for phage-mediated bioremediation

The theoretical use of phages centers on leveraging their ability to precisely manipulate bacterial communities to enhance the biodegradation of APIs, a significant component of the pharmaceutical pollution problem (43). (i) Modulating Microbial Communities: A proposed pathway involves using specific phage cocktails to suppress bacterial strains that compete with or inhibit the growth of known API-degrading microbes. This strategic “weeding” of the microbial community could, in theory, enrich populations that enhance the breakdown of persistent drug residues, potentially complementing existing treatment technologies (42). (ii) Engineered Phages as Delivery Vectors: A more advanced concept involves using engineered phages as vectors to deliver and express key biodegradation genes (e.g., for enzymes like cytochrome P450) directly into environmental bacterial hosts. This would conceptually transform the host into a transient, self-replicating bioremediation unit, offering a novel approach to a persistent issue (41).

### 4.2 Phage-based biosensing: a theoretical framework

The high specificity of phages also makes them ideal theoretical candidates for monitoring pharmaceutical pollution. One could envision a biosensor system where a phage, engineered with a reporter gene, infects a host bacterium that is itself engineered to activate a genetic circuit in the presence of a specific API. The resulting signal would provide a biological detection method for trace-level contamination, although this remains a conceptual framework from synthetic biology.

### 4.3 A critical feasibility and risk analysis

The leap from these compelling concepts to practical application is hindered by significant, and perhaps prohibitive, hurdles that must be addressed (41).

- Technical and Ecological Hurdles:
  - Delivery and Persistence: Maintaining a functionally stable population of phages in a complex, open environment is a monumental challenge. Phages are susceptible to inactivation by sunlight, adsorption to particles, and dilution, far from the controlled conditions of a laboratory or wastewater treatment plant (42).
  - Host Range and Resistance: The narrow host range of most phages necessitates custom cocktails for each unique environment. Furthermore, bacteria rapidly evolve resistance, rendering a static phage treatment ineffective and requiring continuous, unsustainable updates.
  - Unintended Consequences: The deliberate manipulation of microbial communities is ecologically risky. Removing or altering one population can have cascading, unpredictable effects on ecosystem function and resilience, potentially exacerbating ecological damage (41).
- Regulatory and Social Hurdles: The intentional release of engineered or exogenous phages into the environment represents a significant regulatory gray area. Public acceptance of “virus release” for remediation is another major, and often underestimated, barrier to adoption (41).

In conclusion, while environmental virology presents a fascinating frontier for addressing the global issue of pharmaceutical pollution (39, 40), its proposed tools are currently conceptual. The path forward requires extensive fundamental research to overcome the stark technical and ecological challenges (42) and to establish a robust risk-assessment framework before any real-world application can be responsibly considered (41).

## 5 Emerging concerns

The efficacy of antiviral therapies is challenged by the rapid evolution of viruses, which enables the emergence of drug resistance. High replication rates, low-fidelity polymerases, and substantial genetic diversity, particularly in RNA viruses, allow rapid adaptation to selective pressures imposed by therapeutics (44, 45).

### 5.1 Mechanisms of antiviral resistance

Antiviral resistance emerges through multiple molecular mechanisms that are amplified by environmental pharmaceutical pollution. The primary driver is the high mutation rate of RNA viruses, which lack proofreading mechanisms during replication, resulting in rapid genetic diversification (44, 45). While some viruses, such as coronaviruses, encode an ExoN proofreading activity (nsp14) that lowers overall mutation rates, this does not eliminate the generation of resistant variants (46).

Target-site mutations are the most common mechanism. Changes in the viral protein targeted by the drug reduce drug binding affinity or inhibit functional blockage. Examples include:

- RdRp mutations conferring resistance to remdesivir and molnupiravir in SARS-CoV-2.
- Mpro mutations reducing susceptibility to nirmatrelvir (Paxlovid®).
- Reverse transcriptase mutations conferring resistance to NRTIs/NNRTIs in HIV.
- Neuraminidase mutations in influenza, such as H274Y, reducing oseltamivir efficacy (44, 46).

Recombination and reassortment represent an additional mechanism. Co-infection of a host cell with multiple viral strains can lead to genetic exchange, potentially combining resistance mutations from different variants (15, 16). This is particularly relevant in environmental settings where diverse viral populations co-exist and are exposed to sub-therapeutic drug concentrations.

Host-mediated selection occurs when sub-therapeutic concentrations of antivirals select for resistant mutants without fully suppressing viral replication, allowing resistance to become established in the environmental viral community (45). Under drug pressure, pre-existing or *de novo* resistant variants gain a fitness advantage and can dominate viral populations. Although resistance mutations often incur a fitness cost, compensatory mutations can restore viral fitness without compromising resistance, stabilizing resistant lineages and posing persistent clinical and epidemiological challenges (44).

### 5.2 Environmental drivers of antiviral resistance

Beyond clinical settings, environmental reservoirs are increasingly recognized as amplifiers of antiviral resistance within a One Health framework (45). The environmental dimension adds a critical layer of complexity to resistance evolution because antiviral residues in wastewater, surface water, and sediments create chronic selection pressure that can sustain resistant variants even in the absence of clinical use.

Major contributors include:

Wastewater discharge: A proportion of administered antivirals is excreted unmetabolized. Compounds such as oseltamivir carboxylate resist conventional wastewater treatment, entering rivers, lakes, and estuaries at μg/L concentrations during periods of high use, generating sub-therapeutic selective pressure on environmental viral communities (45, 46). This is particularly concerning for influenza viruses in waterfowl, which are natural reservoirs and have been shown to develop oseltamivir resistance after exposure to environmental drug levels (30).

Wildlife exposure: Chronic environmental exposure can select resistant viruses in animal reservoirs, creating the potential for spillback into human populations (45). The potential for environmental selection to act as a resistance reservoir that can re-enter human populations is a significant public health concern.

Industrial effluent: Pharmaceutical manufacturing discharge can contain exceptionally high active ingredient concentrations, forming localized hotspots that accelerate resistance evolution in microbial and viral communities (44).

Sub-therapeutic concentrations: A critical environmental driver is the presence of drugs at concentrations below the therapeutic threshold. These levels do not fully suppress viral replication but exert sufficient selective pressure to favor resistant variants, allowing resistance to establish and persist in environmental reservoirs (28, 47).

### 5.3 Implications and mitigation strategies

Environmental antiviral resistance represents a growing threat to global public health. Effective mitigation requires multi-scale interventions:

- *Enhanced surveillance*: Continuous genomic monitoring of viral populations in clinical, environmental, and wildlife reservoirs for early detection of emerging resistant variants (48).
- *Rational drug use*: Robust stewardship programs in human and veterinary medicine to reduce selective pressures driving resistance (46).
- *Advanced wastewater treatment*: Upgrading WWTPs with tertiary technologies such as ozonation, advanced oxidation processes (AOPs), and activated carbon filtration effectively removes persistent pharmaceutical residues, reducing environmental release and limiting resistance selection (49).

While these technologies can achieve moderate to high removal efficiency for many active pharmaceutical ingredients, they face significant challenges including high energy costs, byproduct formation, membrane fouling, and land requirements (49). Their effectiveness against viral contaminants and resistance gene dissemination remains an area requiring further investigation (44).

Furthermore, the pharmaceutical industry must embrace a forward-looking R&D paradigm focused on developing next-generation antivirals. Future drug discovery efforts must prioritize compounds designed with inherently high genetic barriers to resistance, novel and unexploited mechanisms of action, and, where possible, broad-spectrum activity. This pre-emptive design strategy is crucial for staying ahead of viral evolutionary adaptation (44). The environmental amplification of antiviral resistance exemplifies the pharmaceutical-virology nexus in action. Pharmaceutical pollution creates selective pressures that shape viral evolution outside clinical settings, while the resulting resistant variants can persist in environmental reservoirs and potentially re-enter human populations. This bidirectional flow from human use to environmental exposure to potential spillback demonstrates the interconnectedness of human, animal, and environmental health. The nexus framework highlights that antiviral resistance cannot be understood solely through clinical or laboratory studies; it requires an integrated environmental perspective that recognizes wastewater, wildlife, and industrial effluent as active sites of resistance evolution and dissemination.

Ultimately, the siloed application of these measures will be insufficient. Effectively mitigating the environmental propagation of antiviral resistance mandates a fully integrated One Health strategy. This approach coordinates surveillance, research, policy, and intervention across the interconnected domains of human, animal, and environmental health, recognizing that the health of each is inextricably linked to the others (50).

## 6 Case studies

### 6.1 COVID-19 and environmental pharmaceutical traces

The COVID-19 pandemic served as a large-scale, global case study, dramatically amplifying the long-standing issue of pharmaceutical pollution in aquatic ecosystems and revealing the profound environmental ramifications of a public health crisis (51, 52). This period provided critical insights into the interconnectedness of human health, pharmaceutical consumption, and ecosystem integrity.

The primary cause of this impact was the unprecedented surge in pharmaceutical consumption. Pandemic response protocols triggered a massive global increase in the use of both repurposed drugs such as the antibiotic azithromycin and the antiviral combination lopinavir/ritonavir and established therapeutics for managing severe symptoms, most notably corticosteroids like dexamethasone and prednisolone (53). The widespread and often prophylactic use of these medications placed a new and potent chemical burden on wastewater systems.

This burden was met with inherent inefficiencies in conventional wastewater treatment. Standard wastewater treatment plants (WWTPs) are largely ineffective at removing many complex pharmaceutical compounds. Studies conducted during this period confirmed that removal rates for many of these drugs were consistently below 30%, leading to the substantial and continuous discharge of bioactive residues into receiving rivers and lakes (53). This inefficiency resulted in measurably elevated environmental concentrations of these pharmaceuticals in surface waters across the globe. This phenomenon demonstrated a direct and observable correlation between public health interventions and a distinct signature of environmental chemical pollution (53).

The constant, high-volume discharge of these compounds, even those with relatively short individual half-lives, led to a state of pseudo-persistence in aquatic environments. This ensured continuous, chronic exposure for non-target aquatic organisms, negating any natural attenuation processes (53). Furthermore, the contamination pathway expanded beyond aquatic systems to terrestrial environments. Practices such as irrigating crops with treated wastewater and applying biosolids (sewage sludge) as agricultural fertilizer became significant vectors for transferring pharmaceutical residues into soils, thereby introducing these bioactive compounds into agricultural systems and creating potential pathways for human re-exposure and impacts on soil health (54).

A particularly concerning aspect was the creation of complex contaminant mixtures. The co-occurrence of a wider array of pharmaceuticals with other pervasive environmental contaminants, such as heavy metals, raised significant concerns about the potential for additive, antagonistic, or synergistic toxicological effects on ecosystems, which remain largely unknown and difficult to predict (53).

Beyond ecological impacts, wastewater analysis also functioned as a societal mirror. Wastewater-based epidemiology (WBE) revealed that changing pharmaceutical profiles in sewage quantitatively reflected behavioral and mental health shifts within populations. Notably, increased detection of psychotropic drugs, including antidepressants and anxiolytics, provided a stark, population-level biomarker for the pandemic’s detrimental impact on global mental health (55).

### 6.2 Environmental fate and migration of vaccine nanoparticles

The unprecedented global deployment of nanoparticle (NP)-based vaccines, particularly lipid nanoparticles (LNPs), has introduced a novel class of engineered materials whose potential environmental impact requires scrutiny (56). While their medical benefits are clear, their environmental fate, specifically their migration and transformation, remain a critical knowledge gap. Based on the known properties of these NPs and principles of environmental nanotoxicity, we can forecast their likely behavior.

#### 6.2.1 Composition and release pathways

A diverse array of nanoscale platforms has been employed, including lipid and polymeric nanoparticles designed for antigen delivery and adjuvanted (57). LNPs, the primary vehicle for mRNA vaccines, are complex structures whose stability is engineered for medical use, not environmental persistence (58, 59). A primary pathway for environmental release is through wastewater, arising from the potential excretion of components or metabolites from vaccinated individuals and the improper disposal of medical waste (56).

#### 6.2.2 Predicted transformation and migration

Once in the environment, these NPs will be subject to transformation processes that control their migration. (i) *In Aquatic Systems:* The PEG-lipid coating used to stabilize LNPs (58) is susceptible to degradation, which could lead to particle aggregation, especially in the presence of ions in wastewater or surface water. Aggregated particles will subsequently be sedimented, transferring from the water column to sediment. The lipid bilayers are also likely vulnerable to hydrolysis and photodegradation, potentially leading to the release of their mRNA payload. (ii) *In Sediments and Soils:* Sediments are predicted to be a major sink. Their behavior in soil will be governed by attachment to soil particles and organic matter, strongly limiting their mobility and leading to accumulation near points of entry, such as from wastewater irrigation or landfill leachate.

#### 6.2.3 Current safeguards and regulatory context

It is essential to contextualize these potential environmental fate scenarios within the existing safety framework governing vaccine development and deployment. Several critical factors mitigate immediate environmental risks:

- Replication-Deficient Design: Unlike live viral vaccines, mRNA vaccines encapsulated in LNPs contain no infectious components. The mRNA payload is a transient genetic message that degrades rapidly outside the body and cannot replicate or integrate into host genomes (58, 59).
- Biodegradable Composition: The lipid components of LNPs are designed with biodegradability in mind, utilizing ester bonds that are susceptible to hydrolysis in biological and environmental systems. This intrinsic property, while engineered for human safety, also limits their long-term environmental persistence (58).
- Regulatory Oversight: Vaccine manufacturing and administration are subject to stringent regulatory oversight. Agencies such as the EMA, FDA, and WHO mandate Good Manufacturing Practices (GMP) that include guidelines for the containment and disposal of clinical waste, reducing the likelihood of large-scale environmental release (56).
- Pandemic-Scale Considerations: Despite these safeguards, the unprecedented scale of COVID-19 vaccination campaigns raises legitimate questions about the cumulative environmental load of these novel materials. Current disposal practices for used vials, syringes, and personal protective equipment, while regulated, may not have been designed for the volume generated during a pandemic, highlighting an area where post-market environmental monitoring could be valuable (56).

Thus, while the intrinsic design features of vaccine nanoparticles and existing regulatory frameworks provide significant safeguards against acute environmental risks, the long-term, low-level presence of these materials in wastewater and sediments remains an understudied area warranting continued scientific attention.

#### 6.2.4 Intersection with environmental virology

The environmental fate of vaccine NPs creates a unique nexus with virology. The potential release of protected mRNA and the persistence of synthetic lipid particles in wastewater and sediments could pose unforeseen risks, such as potential interference with natural microbial communities or wastewater treatment processes (56).

In summary, while direct empirical evidence is still emerging, a mechanistic analysis suggests the environmental journey of vaccine nanoparticles will be defined by aggregation, sedimentation, and chemical degradation. This forecast underscores the urgent need for targeted research to understand the ecological implications of this new class of pollutants.

Evidence status: It is important to clarify that the environmental fate scenarios described above are based on mechanistic extrapolation from the known physicochemical properties of lipid nanoparticles and principles of environmental nanotoxicology (56, 58, 59). To date, no direct empirical evidence exists documenting the environmental release, persistence, or ecotoxicological effects of vaccine nanoparticles in real-world aquatic or terrestrial systems. The potential for mRNA release, particle aggregation, or interference with wastewater treatment processes remains speculative and represents a critical knowledge gap requiring targeted empirical investigation. As such, the conclusions drawn in this section should be interpreted as hypotheses derived from mechanistic reasoning rather than established environmental outcomes.

## 7 Key environmental concerns and mitigation frameworks

The case studies presented in the previous section elucidate a critical triad of emerging concerns at the intersection of the pharmaceutical industry and environmental virology: (i) The amplification of antiviral resistance, (ii) The surge of pandemic-related pharmaceutical traces, (iii) The potential ecological implications of novel vaccine nanotechnology.

**Figure 3:**
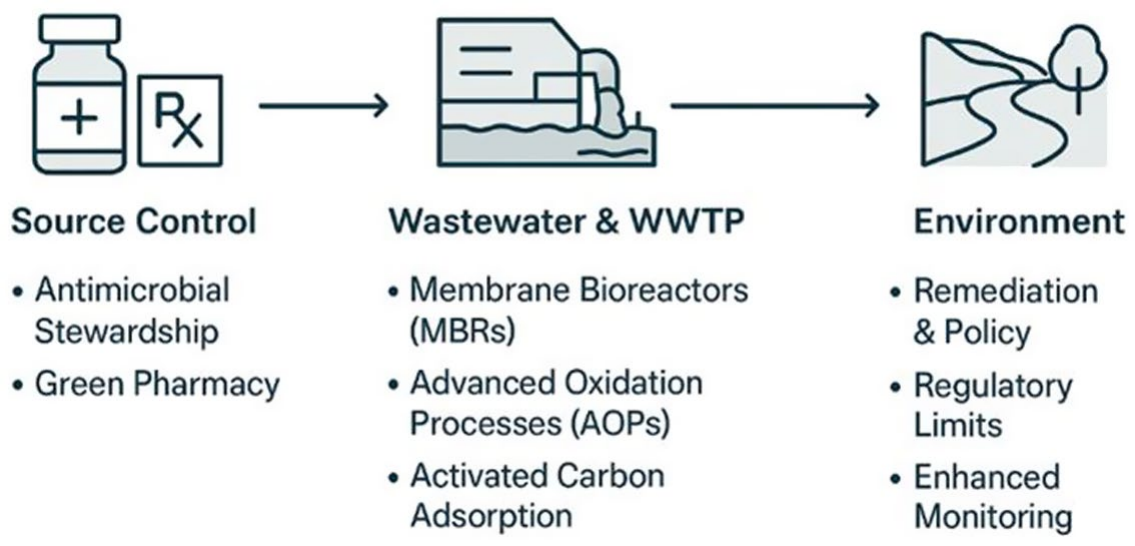
Mitigation strategies across the pharmaceutical lifecycle concept: A horizontal “source-to-sea” timeline showing intervention points.

Effective mitigation of pharmaceutical-viral impacts requires a comprehensive, multi-barrier approach spanning the entire pharmaceutical lifecycle. Figure 3 outlines the strategic intervention points from source control to environmental remediation, highlighting the complementary nature of different mitigation strategies.

Each issue represents a distinct yet interconnected challenge where a pharmaceutical-driven intervention generates complex environmental feedback loops. A comparative analysis of primary environmental concerns reveals distinct mechanistic pathways and mitigation needs. Antiviral resistance arises through mutation and selective pressure driven by the discharge of antivirals in wastewater, requiring interventions such as advanced oxidation processes and antimicrobial stewardship. COVID-19 pharmaceutical traces demonstrate pseudo-persistence due to surges in consumption and inefficient removal in wastewater treatment plants, necessitating source control and wastewater-based epidemiology. Vaccine nanoparticles present unknown ecotoxicological effects due to improper disposal of medical waste and excretion, highlighting the need for improved waste management and green nano-design.

This comparative framework highlights the necessity of moving beyond siloed approaches and adopting integrated, pre-emptive risk management strategies that encompass the entire lifecycle of pharmaceutical products, from design and use to disposal.

## 8 Environmental risk assessment for pharmaceuticals and viral vectors

The established paradigm for pharmaceutical Environmental Risk Assessment (ERA) has traditionally focused on evaluating the direct ecotoxicological effects of individual chemical compounds. However, this framework is critically inadequate for addressing the complex interactions between pharmaceuticals, viral ecology, and the novel challenges posed by biopharmaceuticals like gene therapy viral vectors (60, 61).

### 8.1 The inadequacy of traditional ERA for antimicrobials

A major challenge is that while measured environmental concentrations (MECs) of antimicrobials in aquatic systems are typically orders of magnitude lower than clinical doses, a growing body of evidence shows that these sub-inhibitory concentrations can still exert selective pressure. This pressure enriches antimicrobial-resistant bacteria (ARB) and promotes the horizontal gene transfer of antimicrobial resistance genes (ARGs), a process in which bacteriophages can act as key vectors, directly linking pharmaceutical pollution to changes in viral ecological function (60, 61).

### 8.2 Ecological risks of gene therapy viral vectors: a deep dive

The need for a more sophisticated ERA is critically underscored by the rise of novel biopharmaceuticals, particularly gene therapies that rely on viral vectors (e.g., lentiviruses, adeno-associated viruses). While engineered for human therapeutic use, their potential release into the environment through patient excretion or improper waste disposal poses unique and poorly understood ecological risks that extend far beyond those of chemical APIs (61). These risks necessitate a dedicated assessment framework.

#### 8.2.1 Current safeguards and regulatory oversight

Before examining potential risks, it is essential to contextualize them within the rigorous safety framework governing gene therapy products. Several key features mitigate immediate environmental concerns:

- Replication-Deficient Design: The viral vectors used in approved and investigational gene therapies are engineered to be replication-deficient. Essential genes required for viral replication are deleted, rendering the vector incapable of producing new viral particles on its own. For example, adeno-associated virus (AAV) vectors retain only the inverted terminal repeats (ITRs), with all viral coding sequences removed and supplied *in trans* during manufacturing. Similarly, lentiviral vectors are pseudo typed and lack critical accessory genes, making them dependent on packaging cell lines for production (61).
- Inherent Biological Barriers: Even if a vector were to encounter a wild-type virus, the likelihood of recombination restoring full replication competence is extremely low. Multiple, spatially separated deletions make a single recombination event insufficient to regenerate a wild-type genome. Furthermore, the tropism of these vectors is often altered (pseudo typed) for human cell targeting, which may not be efficient in environmental bacteria or non-target organisms.
- Regulatory Oversight and Containment: Gene therapy products are subject to some of the most stringent regulatory oversight in biomedicine. Agencies such as the FDA and EMA require detailed environmental risk assessments as part of marketing authorization applications. Clinical trial protocols mandate specific containment and disposal procedures for patient excreta and medical waste, significantly reducing the probability of environmental release (61).
- Post-Market Surveillance: As these therapies gain approval, regulatory bodies are increasingly requiring post-market environmental monitoring plans to detect any unforeseen ecological impacts, ensuring a proactive rather than reactive approach to environmental safety.

Despite these substantial safeguards, the theoretical risks outlined below warrant ongoing scientific vigilance, particularly as the scale of gene therapy deployment expands globally.

#### 8.2.2 Potential ecological risk pathways

- Recombination with Wild Viruses: A primary ecological concern is the potential for recombination between the engineered viral vector and circulating wild-type viruses in the environment. Even replication-deficient vectors contain homologous sequences with their wild-type counterparts. A recombination event could theoretically generate a novel, replication-competent virus with altered tropism, pathogenicity, or environmental stability, with unpredictable consequences for wildlife and ecosystem health. However, as noted above, the multiple deletions engineered into modern vectors make this a highly improbable event.
- Horizontal Gene Transfer to Environmental Microbes: Viral vectors are designed for efficient gene delivery. In an environmental context, they could potentially transduce non-target organisms, including environmental bacteria. The insertion of therapeutic transgenes (e.g., for growth factors, immunomodulators) or the vector’s own genetic regulatory elements into a soil or water bacterium could disrupt its physiology, alter its ecological role, or even provide a selective advantage, thereby perturbing microbial community structure and function.
- Persistence and Long-Term Selective Pressure: The capsids of many viral vectors, such as AAVs, are renowned for their physical stability, which is a desired pharmaceutical property but an environmental concern. These vectors could persist for extended periods in water, sediments, or soils. Their continued presence represents persistent, low-level pressure for the selection of microbial communities that can interact with or resist these vectors, potentially enriching for novel viral receptors or defense mechanisms in environmental bacteria.
- Impacts on the Environmental Virome: The introduction of a high concentration of a single, engineered viral genotype into an ecosystem could competitively exclude natural viral populations or alter host-virus dynamics. This could reduce viral diversity and disrupt the “viral shunt,” a key process in nutrient cycling, with cascading effects on microbial and higher trophic levels.

#### 8.2.3 Balancing risk and reality

In summary, while gene therapy viral vectors present theoretical ecological risks that warrant careful study, these must be balanced against the substantial safety features engineered into their design and the rigorous regulatory frameworks governing their use. The replication-deficient nature of these vectors, combined with stringent containment protocols, renders the probability of significant environmental impact exceedingly low under current conditions. Nevertheless, the rapid expansion of gene therapy and the potential for future vectors with novel properties underscore the importance of continued research into their environmental fate and the development of standardized, post-market monitoring protocols. This balanced perspective ensures that regulatory science keeps pace with therapeutic innovation, safeguarding both human health and ecological integrity.

Evidence status: It is essential to distinguish between the theoretical ecological risks described above and the current state of empirical evidence. The potential for recombination, horizontal gene transfer, persistence, and virome disruption are theoretical concerns derived from the known properties of viral vectors and principles of environmental microbiology (61). To date, no documented cases of environmental release, ecological impact, or recombination with wild-type viruses have been reported for approved gene therapy products. The replication-deficient design and stringent regulatory oversight provide substantial safeguards that currently render these risks highly improbable under real-world conditions (61). However, as the scale of gene therapy deployment expands, continued vigilance and post-market environmental monitoring are warranted. The conclusions in this section should therefore be interpreted as hypotheses and risk scenarios rather than established environmental effects.

### 8.3 Toward a next-generation ERA framework

Accordingly, there is an urgent need to establish a standardized, next-generation ERA framework that integrates classical ecotoxicology with molecular and genomic tools. This framework must specifically be equipped to evaluate both the selective potential of antimicrobials and the complex ecological trajectory of viral vectors. Key components should include: (i) *Pre-Release Persistence and Inactivation Studies:* Mandating data on vector stability under various environmental conditions (UV exposure, pH, temperature). (ii) *Recombination and Transduction Assays:* Developing standardized and microcosm-based protocols to assess the potential for genetic exchange with model wild-type viruses and environmental microbes. (iii) *Microcosm and Mesocosm Impact Studies:* Evaluating the effects of vector introduction on microbial community diversity, function, and viral dynamics in controlled environmental simulations. Implementing such an advanced, holistic ERA is indispensable for strengthening global pharmaceutical stewardship and proactively safeguarding ecological and human health security in the face of these emerging biotechnologies (60, 61).

### 8.4 Global perspectives on pharmaceutical pollution and viral implications

The environmental challenge of pharmaceuticals is a global issue, yet its manifestation and intensity vary significantly by region. Incorporating findings from diverse geographical regions is crucial for a comprehensive understanding. The following section highlights key regional case studies that illustrate specific aspects of pharmaceutical pollution with direct relevance to the themes of this review.

#### 8.4.1 Discussion of regional cases

- The European Case (Sulfamethoxazole in Italy): The study by Patrolecco et al. (2018) (62) on Italian rivers underscores that antibiotic persistence is a widespread issue. The slow degradation of sulfamethoxazole ensures continuous selective pressure, favoring bacteria carrying ARGs. This creates stable conditions where bacteriophages can continually mobilize these genes, contributing directly to environmental resistance.
- The Latin American Case (Diclofenac in Mexico): Hernández-Zamora et al. (2025) (63) demonstrate that diclofenac’s impact is pervasive across ecosystem trophic levels. This broad ecotoxicity suggests that viral hosts at multiple levels could be physiologically compromised. A stressed, immuno-disrupted host community presents a more susceptible environment for viral propagation, linking general ecotoxicity directly to virological risk.

The environmental persistence of sulfamethoxazole in Italian rivers (62) demonstrates that antibiotics maintain long-term selective pressure for resistance genes, sustaining a reservoir of mobile genetic material available for bacteriophage-mediated transduction. Similarly, diclofenac contamination in Mexican aquatic systems produces toxic effects across multiple trophic levels (63), suggesting that multi-trophic physiological stress can compromise host health and potentially increase host susceptibility to viral infections.

In conclusion, these regional case studies from Europe and Latin America provide concrete evidence that the nexus between pharmaceuticals and viral ecology is a tangible issue in diverse global ecosystems. A next-generation environmental risk assessment framework must be capable of incorporating such geographically specific data.

### 8.5 The pharmaceutical-virology nexus: a unified framework

This review has synthesized evidence across pharmacology, environmental science, and virology to establish the pharmaceutical-virology nexus as a distinct and consequential field of inquiry. The direct “drug-to-virus” pathway where pharmaceuticals interact with viral particles to alter stability or infectivity and the indirect “drug-to-host-to-virus” pathway where drug-induced stress on bacterial hosts triggers prophage induction and viral community shifts are not separate phenomena but interconnected components of a unified ecological framework.

The nexus concept is more than a descriptive term. It captures the reciprocal and synergistic nature of these interactions: pharmaceutical pollution alters viral ecology, which in turn influences the environmental fate and evolution of pharmaceutical resistance. This dynamic is evident in the amplification of ARG dissemination through phage-mediated transduction, where antibiotics select for resistance while simultaneously promoting the viral vectors that spread it (26). Similarly, the selection for antiviral resistance in environmental reservoirs, such as oseltamivir-resistant influenza in waterfowl, demonstrates how drug release can create persistent resistance pools that may re-enter human populations (30).

What distinguishes this nexus from a simple collection of drug-virus observations is its systemic nature. The pharmaceutical-virology nexus operates across scales from molecular interactions at the capsid surface to population-level shifts in microbial community structure, and ultimately to public health outcomes. Key evidence supporting this systemic view includes:

- Direct molecular interactions: Demonstrating that non-antibiotic pharmaceuticals can bind to viral proteins and alter infectivity (29, 30).
- Indirect host-mediated effects: Antibiotics and other drugs induce bacterial stress responses that trigger prophage induction, fundamentally reshaping viral communities and facilitating gene transfer (15, 16, 26).
- Environmental persistence: The detection of pharmaceutical residues at biologically active concentrations in global water bodies confirms that these interactions are ecologically relevant (64–66).
- Emerging biopharmaceuticals: Vaccine nanoparticles and gene therapy viral vectors represent a new class of pollutants whose environmental fate and ecological risks remain largely uncharacterized (56, 61).

While the field is young and evidence gaps remain, the existing body of work consistently points to a fundamental interconnection that demands a paradigm shift in how we evaluate pharmaceutical pollution. The proposed next-generation Environmental Risk Assessment (ERA) framework, which integrates viral ecological outcomes, provides a pragmatic pathway for translating this conceptual framework into environmental management and regulatory practice. By explicitly incorporating viral dynamics, this framework moves beyond traditional chemical-focused models to address the full spectrum of ecological and public health risks posed by pharmaceutical pollution.

The regional case studies from Italy and Mexico (62, 63) further reinforce this nexus by demonstrating that these interactions are not theoretical but are playing out in diverse global ecosystems. The persistence of sulfamethoxazole in Italian rivers and the multi-trophic ecotoxicity of diclofenac in Mexican aquatic systems illustrate how pharmaceutical pollution creates conditions that can alter viral ecology and host susceptibility across different geographical and ecological contexts. These examples underscore the need for geographically informed, nexus-based approaches to environmental risk assessment and management.

## 9 Synthesis and future research directions: from mechanistic gaps to ecological solutions

The preceding sections have established the pharmaceutical-virology nexus as a unified framework for understanding how pharmaceutical pollution influences viral ecology through direct and indirect pathways. This framework synthesizes disparate evidence from ecotoxicology, virology, pharmacology, and environmental science into a coherent conceptual model. However, to advance this field from a conceptual framework to a predictive science, future research must bridge critical gaps in methodology, mechanism, and ecological translation. The following research priorities are proposed to address the outstanding scientific questions and inform effective mitigation.

This review has synthesized emerging evidence for the pharmaceutical-pollution-viral-ecology nexus. To advance this field from a conceptual framework to a predictive science, future research must bridge critical gaps in methodology, mechanism, and ecological translation. The following research priorities are proposed to address the outstanding scientific questions and inform effective mitigation.

### 9.1 Elucidating mechanistic pathways: beyond direct activity

A primary gap is the detailed molecular mechanism by which diverse pharmaceuticals influence viruses and microbiomes. Future work must move beyond correlation to establish causation. (i) *For non-antimicrobial drugs*: Research should focus on how psychoactive (e.g., SSRIs), metabolic (e.g., metformin), and anti-inflammatory drugs reshape the gut and environmental microbiome. Meta transcriptomic and metabolomic approaches can link these shifts to changes in viral abundance, prophage induction rates, and the expression of viral auxiliary metabolic genes (AMGs) (67). The key question is: do these drugs create dysbiotic states that inadvertently expand viral niches or increase host susceptibility? (ii) *For transformation products and mixtures*: Environmental photodegradation and metabolism create transformation products with unknown bioactivity. Research must employ effect-directed analysis (EDA) coupled with viral infectivity assays to identify which compounds or synergistic mixtures in “pharmaceutical cocktails” are drivers of viral ecological change (68).

### 9.2 Methodological innovations for environmental virology

Current methods are often inadequate to capture the dynamics of this nexus. (i) *Viral Persistence and Infectivity:* Standard qPCR detects viral genetic material but not infectivity. Integrated approaches combining propagation-based methods (e.g., plaque assays for culturable phages) with molecular viability assays (e.g., qPCR) are needed to distinguish intact, infectious viruses from non-infectious debris in pharmaceutical-exposed environments (69). (ii) *Linking Pharma, Microbiome, and Virome:* Multi-omics integration is essential. Study designs should concurrently apply *LC–MS/MS* (for pharmaceutical quantification), *16S/18S rRNA gene sequencing* (for microbiome), and *viral metagenomics (viromics)* to the same environmental samples. This allows for direct statistical modeling of the relationships between pharmaceutical concentration, microbial community structure, and viral community composition/function (70).

### 9.3 Ecological cascades and the role of higher organisms

The ultimate consequences manifest at the ecosystem level, involving higher organisms as reservoirs, amplifiers, and victims. (i) *Wildlife as Reservoirs:* Research must investigate how chronic, low-level pharmaceutical exposure in wildlife (e.g., fish, amphibians, birds) alter their *gut virome and microbiome*, potentially turning them into reservoirs for novel or drug-resistant pathogens. This is a critical link for understanding zoonotic and epizootic disease emergence (71). (ii) *Trophic Transfer and Food Web Disruption:* Studies are needed to trace how pharmaceuticals and their induced changes in microbial/viral communities transfer through food webs (72, 74). Does pharmaceutical stress on primary producers (algae) or decomposers (bacteria) cascade upward, affecting invertebrate and vertebrate health through nutritional or pathogenic pathways?

### 9.4 Toward proactive mitigation and intervention

Scientific understanding must translate into action. A multi-pronged mitigation strategy is required: (i) *Source Control and Advanced Treatment:* Upgrading wastewater treatment with *advanced oxidation processes (AOPs) or specific adsorbents* (e.g., molecularly imprinted polymers) can target persistent pharmaceuticals. *Wastewater-Based Epidemiology (WBE)* should be expanded to monitor not only human pathogens but also pharmaceutical loads and marker ARGs/viral sequences, providing real-time data for treatment optimization. (ii) *Ecological Risk Forecasting:* Developing *mechanistic models* that integrate pharmaceutical pharmacokinetics, microbial ecology, and viral population dynamics will enable forecasting of ecological risks under different pollution scenarios. These models can guide regulatory decisions on environmental discharge limits for high-risk APIs. (iii) *Green Pharmacy and Circular Design:* A fundamental solution lies in *designing greener pharmaceuticals* with faster environmental degradation and lower ecological impact, without compromising therapeutic efficacy, a true application of the *One Health* principle at the molecular design stage.

By systematically addressing these mechanistic, methodological, and ecological frontiers, the scientific community can transform the “untold nexus” into a well-characterized dimension of planetary health, enabling evidence-based strategies to safeguard ecosystem integrity and public health.

## 10 Conclusion and future perspectives for ecotoxicology

**Figure 4:**
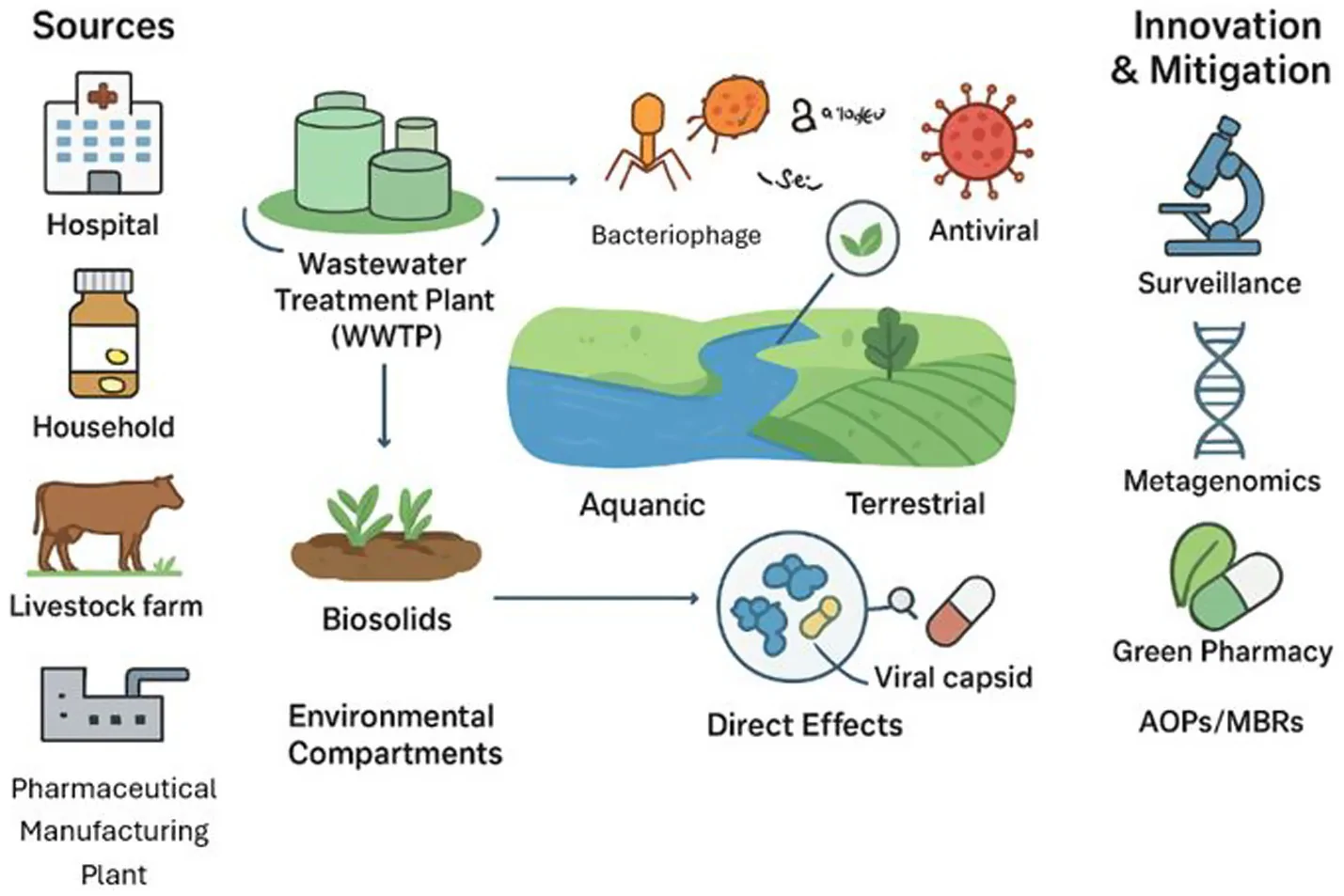
The pharmaceutical-virology nexus: pathways and impacts concept: A central infographic illustrating the complete lifecycle of pharmaceuticals and their interactions with viral ecology.

This review has synthesized a previously overlooked dimension of ecotoxicology: the complex nexus between pharmaceutical pollution and viral ecology. This nexus defined by the bidirectional and synergistic interactions between pharmaceuticals and viral communities represents a paradigm shift in how we understand pharmaceutical pollution and its consequences for ecosystem and human health. From an ecotoxicological perspective, pharmaceutical pollution from active pharmaceutical ingredients (APIs) to vaccine-derived nanoparticles poses multifaceted threats by altering viral stability, infectivity, and community dynamics; accelerating the phage-mediated dissemination of antibiotic resistance genes; and introducing novel entities (e.g., LNPs, viral vectors) into ecosystems with unknown ecological consequences (Figure 4).

A central contribution of this review to ecotoxicology is the novel dual-pathway framework (direct “drug-to-virus” and indirect “drug-to-host-to-virus” interactions), which provides a mechanistic basis for understanding how APIs impact viral communities, a level of biological organization traditionally excluded from environmental risk assessment.

Key challenges for future ecotoxicological research include:

1. The need for advanced ecotoxicological risk assessment (ERA) frameworks capable of addressing sub-lethal effects on viral ecology, including resistance selection at environmentally relevant concentrations.
2. The integration of viral ecological endpoints into standard ecotoxicity testing protocols.
3. The development of methods to distinguish between viral infectivity and mere genetic detection in environmental samples.

The COVID-19 pandemic underscored these vulnerabilities, revealing how shifts in pharmaceutical use translate into measurable environmental impacts on viral communities a finding with direct relevance to ecotoxicological monitoring.

While ecotoxicology provides the core scientific framework, addressing these challenges requires interdisciplinary collaboration. A One Health approach that integrates genomic surveillance, green pharmacy initiatives, advanced wastewater treatment technologies, and circular economy strategies (e.g., drug take-back programs) will be essential to translate ecotoxicological insights into environmental management and public health policy. Expanding wastewater epidemiology to encompass pathogens, chemical pollutants, and resistance markers will further strengthen environmental monitoring efforts.

Ultimately, progress in this emerging frontier of ecotoxicology depends on collaboration among virologists, ecotoxicologists, environmental engineers, and risk assessors. Sustainable pharmaceutical innovation will require proactive drug design and environmental management systems that explicitly account for the interdependence of human, animal, and ecological health ensuring that medical advances are achieved without compromising planetary integrity.
